# Evaluation of Different Normalization and Analysis Procedures for Illumina Gene Expression Microarray Data Involving Small Changes

**DOI:** 10.3390/microarrays2020131

**Published:** 2013-05-21

**Authors:** Daniel M. Johnstone, Carlos Riveros, Moones Heidari, Ross M. Graham, Debbie Trinder, Regina Berretta, John K. Olynyk, Rodney J. Scott, Pablo Moscato, Elizabeth A. Milward

**Affiliations:** 1 Centre for Bioinformatics, Biomarker Discovery and Information-Based Medicine, The University of Newcastle, Callaghan, NSW 2308, Australia; E-Mails: carlos.riveros@newcastle.edu.au (C.R.); regina.berretta@newcastle.edu.au (R.B.); rodney.scott@newcastle.edu.au (R.J.S.); pablo.moscato@newcastle.edu.au (P.M.); liz.milward@newcastle.edu.au (E.A.M.); 2 School of Biomedical Sciences and Pharmacy, The University of Newcastle, Callaghan, NSW 2308, Australia; E-Mail: moones.heidari@uon.edu.au; 3 Discipline of Physiology and Bosch Institute, University of Sydney, Sydney, NSW 2006, Australia; 4 Australian Research Council Centre of Excellence in Bioinformatics, Callaghan, NSW 2308, Australia; 5 School of Electrical Engineering and Computer Science, the University of Newcastle, Callaghan, NSW 2308, Australia; 6 School of Biomedical Sciences, CHIRI Biosciences Research Precinct, Faculty of Health Sciences, Curtin University, Bentley, WA 6102, Australia; E-Mail: rmgraham@curtin.edu.au; 7 School of Medicine and Pharmacology, University of Western Australia, Fremantle, WA 6160, Australia; E-Mail: debbie.trinder@uwa.edu.au; 8 Western Australian Institute for Medical Research, Perth, WA 6000, Australia; 9 Department of Gastroenterology, Fremantle Hospital, Fremantle, WA 6160, Australia; E-Mail: john.olynyk@health.wa.gov.au; 10 Curtin Health Innovation Research Institute, Curtin University, Bentley, WA 6102, Australia; 11 Institute for Immunology & Infectious Diseases, Murdoch University, Murdoch, WA 6153, Australia; 12 The Division of Molecular Medicine, Hunter Area Pathology Service, New Lambton, NSW 2305, Australia

**Keywords:** gene expression microarray, normalization, Illumina

## Abstract

While Illumina microarrays can be used successfully for detecting small gene expression changes due to their high degree of technical replicability, there is little information on how different normalization and differential expression analysis strategies affect outcomes. To evaluate this, we assessed concordance across gene lists generated by applying different combinations of normalization strategy and analytical approach to two Illumina datasets with modest expression changes. In addition to using traditional statistical approaches, we also tested an approach based on combinatorial optimization. We found that the choice of both normalization strategy and analytical approach considerably affected outcomes, in some cases leading to substantial differences in gene lists and subsequent pathway analysis results. Our findings suggest that important biological phenomena may be overlooked when there is a routine practice of using only one approach to investigate all microarray datasets. Analytical artefacts of this kind are likely to be especially relevant for datasets involving small fold changes, where inherent technical variation—if not adequately minimized by effective normalization—may overshadow true biological variation. This report provides some basic guidelines for optimizing outcomes when working with Illumina datasets involving small expression changes.

## 1. Introduction

Microarray studies have been particularly successful for identifying genes with large expression changes in conditions such as cancer. The challenge is to extend microarray technology into robust identification of smaller gene expression changes. This requires array platforms with a high degree of sensitivity and specificity and data analysis tools that generate accurate results. While increasing experimental group sizes can improve the detectability of subtle changes, one major challenge in microarray analysis is the detection of small, but “real”, expression changes in small datasets.

The Illumina microarray platform has become one of the main platforms for “transcriptomic” studies. Each Illumina BeadChip array comprises randomly positioned silica beads, each containing hundreds of thousands of copies of a specific 50-nucleotide probe sequence. On average, each probe is replicated on at least 15 beads randomly distributed across each array. The large number of replicate beads minimizes artefacts that may arise due to intra-array location and other factors and provides a high degree of internal technical replication, facilitating generation of reliable raw data [1,2,3,4,5,6]. 

The technology has performed well in comparative studies of different platforms by the Microarray Quality Control (MAQC) consortium [7,8] and others [9,10,11], but such studies have not provided detailed comparisons of the performance of different data analysis tools. Various open source tools are available to analyse Illumina data, such as *lumi* [12], *limma* [13] and other Bioconductor packages [14], which use the R programming environment. Schmid and colleagues have compared different normalization methods available through the R environment and Illumina’s proprietary software, recommending particular approaches depending on the characteristics of a particular dataset [15]. However this study did not investigate how different differential expression analysis techniques or combinations of normalization strategy and differential expression analysis technique affect final outcomes—there is still little information available on this.

In addition, as Bioconductor packages require knowledge of the R programming language, they are currently used primarily by researchers with stronger computing backgrounds and by more specialized research groups doing large quantities of array analysis. These approaches are less commonly used by researchers doing occasional array studies or performing downstream analyses of array data provided under contract by large facilities or by researchers with restricted computing expertise, as is the case for many graduates from biological disciplines. 

Most novice Illumina microarray users instead rely on established “black box” procedures developed by Illumina and other companies. Therefore, while the Illumina platform appears well-suited to working with datasets involving small expression changes, as described above, the effects of different computational approaches need to be investigated more closely. In this study, we have examined how different normalization and differential expression analysis tools may influence analyses of small, low fold-change datasets on this platform. 

Following initial scanning of BeadChips by Illumina’s BeadScan software, there are three phases of processing of scanned BeadChip data (bead level data): (1) Local background subtraction and averaging of probe replicates generating bead summary data; (2) Transformation and normalization; (3) Analysis of differential expression. The different data processing steps and associated issues are briefly reviewed below.

### 1.1. Generating Bead Summary Data

Initial data pre-processing in the proprietary Illumina GenomeStudio (formerly BeadStudio) software provides users with bead summary data in the form of a single signal intensity value for each probe. This value is calculated by subtracting the local background from the signal intensity for each bead, then taking the mean of all beads containing a given probe. While the *beadarray* package available through R/Bioconductor allows the user to work with raw bead level data [16], these data impose considerable storage requirements and are not yet commonly utilized by novice microarray users. Furthermore, Dunning and colleagues investigated the effects on bead level data of the pre-processing summarization methods used by GenomeStudio and concluded that these are beneficial for reducing bias and robust determination of gene expression [17]. For these reasons, we have restricted the present investigation to bead summary data that have already been generated by pre-processing algorithms in GenomeStudio.

### 1.2. Transformation and Normalization

Raw bead summary intensity values are usually normalized by one or more transforming functions. Reasons for normalizing can include forcing a normal data distribution or increasing comparability between probes, samples, chips, machines or platforms. Even small technical variations (e.g., cRNA loading on arrays, scanning and hybridization inconsistency) can sometimes cause considerable differences in signal intensities. The overarching aim of normalization is to reduce differences due to technical variation (false positives), while conserving true biological effects (*i.e.*, maximizing true positives and minimizing false negatives).

Prior to normalization, it is generally recommended that a correction step be performed to adjust for between-array differences in non-specific signal intensities (*i.e.*, background correction). Using GenomeStudio, this correction involves subtracting the mean signal of negative control probes in a particular array from each bead summary value in that array. While recommended by Illumina, several groups suggest this particular method is flawed [15,17,18] and propose alternative correction approaches available through the Bioconductor project. 

Following background correction (or not), microarray data are generally normalized by one of several different approaches. Here, we have investigated all four optional normalization strategies in the GenomeStudio software: *Average*, *Cubic Spline*, *Quantile* and *Rank Invariant*, as well as the *No Normalization* option. *Average* involves normalization to the mean signal of each sample; *Cubic Spline* and *Quantile* apply different forms of quantile normalization to bead summary data [19,20]; *Rank Invariant* normalizes data based on values of probes that do not change their ranking across samples. In the first section of the study, we have compared the effects of the different GenomeStudio normalization strategies within each of three different analytical approaches.

### 1.3. Analysis of Differential Expression

Following normalization, different analytical approaches are used to identify genes with altered expression between experimental conditions. The challenge for any analytical approach lies in reducing false positives (Type I or α errors), while avoiding false negatives (Type II or β errors). The use of a statistical *p*-value approach allows estimation of false positive error probability, which can be considerable when conducting large numbers of comparisons. Yet, conversely, the methods currently used to adjust for multiple comparisons [21] are often very conservative and may miss real changes. Adjustments of this kind may be most useful for identifying restricted groups of target genes (e.g., class prediction aimed at identifying biomarkers for diagnosis or prognosis). For studies aimed at identifying complete sets of target genes (e.g., class comparison or class discovery aimed at understanding biological mechanisms), accepting non-informative false positives may be less problematic than omitting informative genes. Consequently, minimizing false negatives by not applying a multiple testing correction has been recommended for such studies [22,23]. As our study has focused on approaches suitable for identifying complete sets of differentially expressed genes (class discovery), a multiple testing correction has not been applied to most analyses.

In addition to exploring the effects of different normalization strategies, we have also assessed how outcomes are affected by applying each of three different analytical approaches to the normalized data. Two of the three approaches tested used statistical significance as the inclusion criteria: GenomeStudio differential expression and GeneSpring differential expression. GenomeStudio was assessed because it is the Illumina proprietary software. GeneSpring is a widely-used, commercially available application with a number of features not present in GenomeStudio, including additional statistical capabilities. The third analytical approach assessed was a Max Cover (α,β)-k Feature Set approach (*Max Cover* (*α*,*β*)*-FS*) [24,25,26,27]. Whereas the GenomeStudio and GeneSpring algorithms use the average magnitude and variance of the signal intensity, *Max Cover* (*α*,*β*)*-FS* considers primarily the distribution of the test and control replicates relative to one another and the ability of each probe to discriminate between replicates from different classes (e.g., different experimental conditions). It is not based upon fold-change cut-offs or the statistical significance of comparisons of mean expression measures.

We analysed two comparable Illumina datasets with relatively small expression changes. These were from (i) heart and (ii) brain samples of biological replicates of mice fed a short-term high iron diet and control mice fed a normal diet. Short-term high iron diets cause only modest changes in heart gene expression [28], and our studies suggest changes in the brain are even smaller, possibly because the blood-brain barrier may help protect the brain from high systemic iron [29]. The study examines the effects of applying different normalization and expression analysis approaches to these datasets.

## 2. Experimental Section

### 2.1. Animals

All animal work was approved by the Animal Ethics Committee of the University of Western Australia. Male mice of the AKR strain were fed either normal chow or a high-iron diet (normal chow supplemented with 2% carbonyl iron for three weeks prior to sacrifice). The high-iron regimen used results in significantly higher iron indices and iron loading in the liver [30]. At 10 weeks of age, mice were sacrificed under anaesthesia (50 mg/kg ketamine, 10 mg/kg xylazine), and blood was removed by transcardiac perfusion with isotonic saline. Heart and brain tissue was collected from biological replicates (n ≥ 4 per group), snap-frozen in liquid nitrogen and stored at −80 °C.

### 2.2. Microarray Experiments

Total RNA was isolated using TRI Reagent (Ambion), purified and concentrated using the RNeasy MinElute Kit (Qiagen) and amplified with the Illumina TotalPrep RNA Amplification Kit (Ambion). Gene expression was assessed in biological replicates using Illumina Sentrix MouseRef-8 (v1.1) BeadChip arrays. BeadChips were scanned using Illumina BeadArray reader and BeadScan software. For each tissue, all sample preparation and microarray experimentation was done simultaneously using arrays from the same batch, in order to avoid any potential batch effects. Following quality control assessment of microarray data, one control heart RNA sample was flagged as an outlier and removed from further analysis.

### 2.3. Microarray Data Analysis

#### 2.3.1. Normalization and Differential Expression Analysis

Bead summary data were normalized separately for each dataset (heart, brain), using each of the four normalization procedures (*Average*, *Cubic Spline*, *Quantile* and *Rank Invariant*) available in GenomeStudio v2010.3 (Illumina). Non-normalized data were also examined. The algorithms and parameter settings used to assess differential gene expression were:

(1) GenomeStudio v2010.3—The Illumina Custom algorithm in the GenomeStudio software assesses three components of variation (sequence specific biological variation, non-specific biological variation and technical error). Probes returning a *p* value < 0.05 in comparisons of the control and test classes were considered to be detecting differential expression. A more detailed description is given in the GenomeStudio Gene Expression Module User Guide [31].
(2) GeneSpring GX 11.0 Software—The usual default settings of the GeneSpring program apply further transformation and normalization steps; however, this can introduce substantial artefacts when applied to data already normalized by other approaches. For these reasons, these additional normalization steps were not applied. Differential expression was determined by an unpaired *t* test (*p* < 0.05).
(3) Max Cover (α,β)-k Feature Set Approach—*Max Cover* (*α*,*β*)*-FS* is a multivariate method that selects a set of probes that, as a collective, can discriminate well between the experimental test and control groups [27]. This algorithm consists of a two-stage filter process. Firstly, Fayyad and Irani’s algorithm [32] is used to discretise the data. For each probe, the algorithm orders the samples based on signal intensity and converts continuous data to binary data based on different intensity thresholds. It then selects the threshold that minimizes the class-information entropy of the samples, creating a binary dataset and discards the probes that are not discriminative enough, according to the minimum description length principle (filtering) [27]. Secondly, the algorithm finds a solution for the Max Cover (α,β)-k Feature Set problem [24]. This is achieved by comparing, for each probe, all possible pairs of samples, whether controls or tests, in order to extract an optimal set (solution) of probes (“features”) with both strong inter-class differentiation and strong intra-class similarity [25,26,27]. This approach differs from statistical methods, such as GenomeStudio and GeneSpring, in that instead of only considering means and variance measures, it preserves information about the individual samples within each class. It also identifies solutions involving sets of probes. These solutions reflect interrelationships between different probes—information which is often lost when considering each probe individually.

A subset of analyses were performed in which a multiple testing correction (Benjamini Hochberg False Discovery Rate) was applied to background-subtracted data normalized using the strategies described above and assessed for differential gene expression using GenomeStudio.

In addition to investigating the effects of these normalization strategies and analytical approaches on background-subtracted data, we also investigated the effect of omitting background correction before normalizing data using the four available options (as well as *No Normalization*) and performing differential expression analysis in GenomeStudio.

To compare these various approaches to those available through the Bioconductor project, bead summary data were exported from GenomeStudio and analysed with the Bioconductor packages *limma* [13] and *lumi* [12], using pipelines recommended by the tool creators. For *limma*, this involved invoking the *neqc* function (background correction using a normal-exponential convolution model, quantile normalization and log_2_ transformation) followed by replicate summarization by fitting a linear model and differential expression analysis using moderated *t*-statistics with empirical Bayes’ shrinkage of the sample variances [33]. For *lumi*, this involved background correction using *bgAdjust*, variance stabilizing transformation and robust spline normalization, followed by replicate summarization and differential expression analysis using the *limma* functions described above [12].

#### 2.3.2. Filtering of Non-Specific Probe Signals

**Figure 1:**
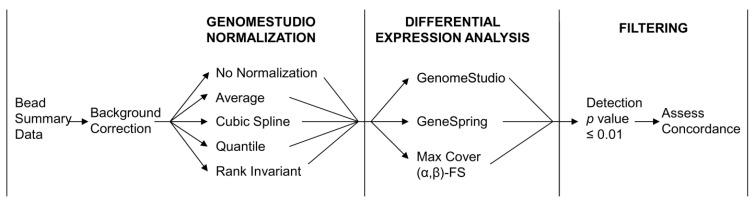
Flowchart illustrating the different normalization procedures and differential expression algorithms used.

To avoid distortion of the results by noise, we removed probes returning signals that were highly likely to be due to non-specific background signal rather than specific probe-target hybridization. The specificity of individual probe signals was estimated using the detection *p*-value, which is the probability of seeing a certain signal level without probe-target hybridization [31]. All probes returning a detection *p*-value > 0.01 (1% false positive rate, as recommended by Illumina) in both the control group and the high iron group were eliminated from further analysis. As illustrated in Figure 1, this step was performed after normalization and differential expression analysis—the GenomeStudio software does not allow the removal of specific probes before normalization and analysis, as might be preferred.

#### 2.3.3. Assessment of Probe Set Concordance

Different combinations of normalization and analysis approaches were applied as detailed in the Results section. The degree of agreement of the resulting probe sets, henceforth termed “concordance”, was calculated as either a number or a percentage. In the first instance, the concordance of two probe sets generated by different normalization strategies or analytical approaches was defined as the number of overlapping probes between the two sets. In the second instance, the concordance was defined as the percentage of overlapping probes calculated against the total number of probes in each particular probe set. Comparable measures, notably number of overlapping genes (NOG) and percentage of overlapping genes (POG), have been used previously to assess outcome concordance [7,34].

In this study, we will be considering concordance in three separate contexts: (1) the concordance between the probe sets generated by the different normalization strategies; (2) the concordance between the probe sets generated by the various types of differential expression analysis approaches; and (3) the concordance between the pathways enriched within each probe set.

#### 2.3.4. Summary of Analysis and Evaluation

A schematic summarizing the different steps in normalization, differential expression analysis and subsequent filtering is given in Figure 1.

### 2.4. Pathway Analysis

The Database for Annotation, Visualization and Integrated Discovery (DAVID [35]) was used to identify enriched pathways in select probe sets [36,37]. The full list of genes included on the array was used as the background list. DAVID organizes gene lists into pathways and identifies those that have an enrichment of differentially expressed genes relative to how many genes would be expected to fall into each pathway by chance alone.

## 3. Results

### 3.1. Comparison of Normalization Methods

#### 3.1.1. Probe Set Generation

For each of the two datasets (heart, brain), a total of 15 probe sets was generated. As summarized in Figure 1, these probe sets were generated by applying each of the four GenomeStudio normalization strategies (*Average*, *Cubic Spline*, *Quantile*, *Rank Invariant*) or the *No Normalization* option to background-corrected data, followed by each of the three analytical approaches (GenomeStudio, GeneSpring, *Max Cover* (*α*,*β*)*-FS*). These probe sets were then filtered to remove probes that returned a detection *p*-value above 0.01 in both conditions in order to eliminate probes at background levels.

**Figure 2:**
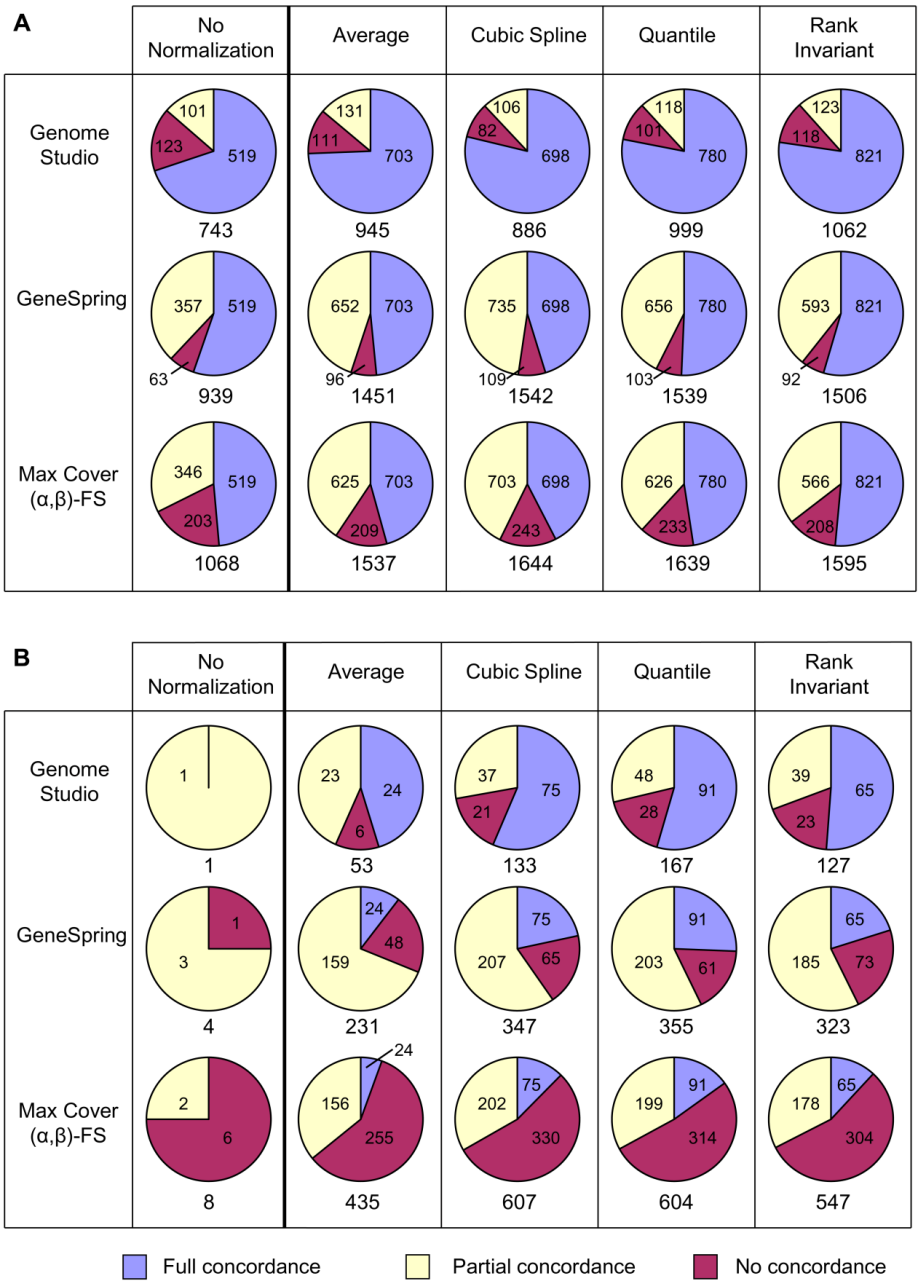
Comparison of concordance between different analytical approaches for each normalization strategy. Concordance of probe sets generated by different analytical approaches was assessed for (**a**) heart array data and (**b**) brain array data. Numbers of fully or partially concordant or discordant probes are shown on the charts, with the total number of probes generated by each combination shown below.

Both datasets showed generally small expression changes (<2-fold), with only 2.1% and 0.4% of changes being over 2-fold in the heart and brain datasets, respectively. Irrespective of the normalization strategy used, probe sets generated from the brain arrays contained a smaller number of probes than those from the heart arrays (Table 1, Figure 2), consistent with fewer gene expression changes in the brain. 

**Table 1:** Concordance in probe sets generated by different normalization strategies. The data are presented as the means of the number of overlapping probes between each possible pairwise comparison of the five normalization strategies, with the means of the percentage overlaps for the same comparisons in parentheses.

	No Normalization	Average	Cubic Spline	Quantile	Rank Invariant
**Heart Dataset**					
GenomeStudio	503 (88.2)	760 (80.4)	738 (83.3)	787 (78.8)	791 (74.5)
GeneSpring	724 (73.6)	1,235 (78.0)	1,374 (78.1)	1,375 (78.3)	1,324 (77.3)
Max Cover (α,β)-FS	781 (71.3)	1,181 (76.8)	1,282 (78.0)	1,278 (78.0)	1,231 (77.1)
**Brain Dataset**					
GenomeStudio	*	44 (82.4)	93 (70.2)	95 (56.9)	85 (67.2)
GeneSpring	*	134 (57.9)	248 (71.5)	248 (70.0)	209 (64.8)
Max Cover (α,β)-FS	*	190 (43.8)	402 (66.3)	401 (66.4)	320 (58.6)

* Excluded from comparisons to avoid bias.

#### 3.1.2. Effects of the Different Normalization Strategies on Probe Set Concordance

In order to determine the influence of normalization on probe set concordance (defined in Section 2.3.3), for each particular analytical approach we performed pairwise comparisons between the different probe sets generated using each of the five normalization strategies. For example, the individual probe sets generated by GeneSpring for each of the five different normalization strategies were compared to each other, giving a total of 10 comparisons. This was also done for each of the other two analytical approaches (GenomeStudio, *Max Cover* (*α*,*β*)*-FS*), giving a total of 30 comparisons.

In general, irrespective of which analytical approach or dataset was used, the *No Normalization* strategy identified relatively small probe sets. In the case of the heart dataset, these were usually highly concordant with the sets identified by other normalization methods (Table 1, Table S1). This suggests that the omission of a normalization step yields fewer false positives, but at the cost of more false negatives, making it less effective than the other strategies for class comparison, although still of possible value for biomarker discovery.

However, in the case of the brain dataset, the use of the *No Normalization* strategy gave extremely small probe sets for all analytical approaches, sometimes containing only a single probe. This grossly distorted the calculations of the concordance between the various normalization strategies for the brain dataset. Therefore, this strategy was not included in the concordance calculations for the brain dataset presented in Table 1.

On average, all four normalization methods (*i.e.*, *Average*, *Cubic Spline*, *Quantile* and *Rank Invariant*) gave comparable levels of concordance; however, the *Average* method produced smaller probe sets with a generally lower mean concordance in the brain dataset (Table 1).

Similar trends were observed when a multiple testing correction was applied to GenomeStudio analysis of the heart dataset, with the *No Normalization* strategy producing far smaller probe sets than the four normalization methods. As for non-corrected data, concordance was high between the different normalization methods, with *Average* producing the smallest probe sets and *Rank Invariant* producing the largest (Table S2). When the multiple testing correction was applied to GenomeStudio analysis of the brain dataset, no probes were identified as having significantly altered expression, irrespective of which normalization strategy was used.

As there have been questions raised in the literature over the suitability of the GenomeStudio background correction procedure [15,17,18], we generated probe lists from non-background corrected data using the five normalization strategies in combination with GenomeStudio differential expression analysis and repeated the pairwise comparisons described above. In almost all cases, omission of background correction gave rise to larger probe sets than those obtained when background correction was applied. Overall, percentage concordance between different normalization methods showed similar trends, whether data were background corrected or not (Table S3).

Next, concordance was assessed across the different analytical approaches.

### 3.2. Comparison of Analytical Approaches

#### 3.2.1. Definition of Concordance for Comparisons of Analytical Approaches

For each particular normalization strategy (including the *No Normalization* strategy), we compared the concordance of the probe sets identified by each of the three different analytical approaches. (This is distinct from the concordance assessed by pairwise comparisons of normalization strategies for a single analytical approach, considered above). For each normalization strategy, a probe was classed as having “full concordance” if it was identified by all three analytical approaches, “partial concordance” if identified by two of the three approaches or “no concordance” if identified by only one approach. 

#### 3.2.2. Effects of the Different Analytical Approaches on Probe Set Concordance

Figure 2 highlights the considerable differences in both numbers and proportions of identified probes that can occur with the various methods. However, some general conclusions can be drawn. For both datasets, the numbers of probes identified when using the *No Normalization* method were much lower than those identified when using each of the four normalization strategies. All four normalization strategies generally produced similar levels of concordance, again with the exception of the *Average* strategy, which produced a lower proportion and number of fully concordant probes in the brain dataset than other strategies (blue sectors, Figure 2). Of the other normalization strategies, overall *Quantile* performed most strongly when considered across both datasets, based on the percentage and number of fully concordant probes. 

When considering analytical approaches, GenomeStudio gave the highest proportion of full concordance (blue sectors, Figure 2). However, this was largely because this approach produced smaller probe sets. GeneSpring generally gave the highest proportion of combined full and partial concordance (blue and yellow sectors, respectively, Figure 2). *Max Cover* (*α*,*β*)*-FS* gave the largest probe sets and, therefore, the greatest number and proportion of discordant probes (red sectors, Figure 2). Some of these may be false positives, but others may be real changes missed by other approaches. This is assessed more fully in the pathway investigations discussed below.

### 3.3. Comparison with Bioconductor Packages

To determine how the results obtained using these approaches compare with those obtained using more flexible, yet computationally-demanding, tools available through the Bioconductor project, data processing and analysis of the heart and brain datasets was undertaken using two Bioconductor tools designed for analysis of Illumina microarrays: *lumi* and *limma*. In the absence of a multiple testing correction, *lumi* and *limma* both generated probe sets that were larger than those generated by any other approach for the heart dataset, and only the *Max Cover* (*α*,*β*)*-FS* approach returned larger probe sets for the brain dataset. The probe sets generated by *lumi* and *limma* were highly concordant with one another (>90% for both heart and brain datasets). For the heart dataset, the *lumi* and *limma* approaches both identified more than 90% of the probes found by all analytical combinations involving *Cubic Spline*, *Quantile* and *Rank Invariant*, with GenomeStudio analyses showing the greatest percentage concordance, though possibly due to the smaller size of GenomeStudio probe sets (Table S4). Concordance was slightly lower for the brain dataset, particularly for combinations involving the *Max Cover* (*α*,*β*)*-FS* approach; however, this may simply reflect the large size of probe sets generated using this method, as described above.

### 3.4. Comparison of Pathway Analysis Outcomes

#### 3.4.1. Definition of Concordance for Comparisons of Enriched Pathways

We next conducted KEGG pathway enrichment analysis using DAVID for the 12 different gene sets generated for each dataset by using each of the four normalization strategies (*Average*, *Cubic Spline*, *Quantile*, *Rank Invariant*) with each of the three analytical approaches (GenomeStudio, GeneSpring, *Max Cover* (*α*,*β*)*-FS*). For each normalization strategy, we determined the number of concordant pathways across the different approaches, where “concordant” denotes pathways common to two or more approaches (Table 2).

**Table 2:** Comparison of outcomes from pathway enrichment analysis. Table displays the total number of pathways identified as enriched in gene lists generated using different combinations of normalization strategies and analytical approaches. Numbers of concordant pathways are shown in parentheses.

Heart Dataset				
	*Average*	*Cubic Spline*	*Quantile*	*Rank Invariant*
GenomeStudio	14 (12)	11 (8)	16 (10)	18 (11)
GeneSpring	24 (22)	18 (16)	16 (13)	18 (17)
*Max Cover* (*α*,*β*)*-FS*	18 (18)	20 (16)	19 (15)	19 (18)
Brain Dataset				
	*Average*	*Cubic Spline*	*Quantile*	*Rank Invariant*
GenomeStudio	0 (0)	2 (2)	3 (2)	3 (3)
GeneSpring	2 (0)	2 (2)	2 (2)	3 (2)
*Max Cover* (*α*,*β*)*-FS*	4 (0)	4 (2)	5 (2)	6 (3)

#### 3.4.2. Effects of Different Normalization and Analytical Approaches on Pathway Analysis

The pathways identified as enriched were strongly affected by both normalization strategy and analytical approach and also varied considerably between the two datasets. For all analytical approaches in both datasets, *Rank Invariant* normalization generally yielded both more pathways and more concordant pathways (Table 2). Unexpectedly, although (as described above) *Max Cover* (*α*,*β*)*-FS* generated probe sets with the most discordant probes (Figure 2), it generally yielded both more pathways and more concordant pathways than the other analytical approaches (Table 2). Of the other two approaches, GeneSpring identified more concordant pathways than GenomeStudio.

#### 3.4.3. Probe Set Concordance and Outcomes of Pathway Analysis

It was observed that approaches that generally show high probe set concordance can still fail to identify pathways of probable importance. One example was the “insulin signalling pathway”. Diabetes is one of the classical triad of symptoms seen at advanced stages of the human iron overload disorder hemochromatosis and iron overload arising due to various causes has been associated with insulin perturbations and type 2 diabetes [38,39]. Furthermore, the insulin signalling pathway has been observed to alter in association with oxidative stress and cell death in other mouse models of iron overload [40,41]. This pathway was identified as significantly enriched in the heart dataset when using all four normalization strategies in combination with the *Max Cover* (*α*,*β*)*-FS* approach (>1.9-fold enrichment, *p* < 0.01). In contrast, approaches that yielded relatively few discordant probes, such as *Quantile* or *Rank Invariant* in combination with GeneSpring, failed to identify this potentially important pathway as significantly enriched. 

Conversely, approaches that generally show high probe set discordance may sometimes identify pathways of potential importance not picked up by other approaches. For example, analysis of gene lists from the heart dataset generated using *Average* normalization with *Max Cover* (*α*,*β*)*-FS*, which had a relatively large number of discordant probes, identified the pathway “acute myeloid leukaemia” (2.6-fold enrichment, *p* = 0.009). This pathway was not detected by other approaches, yet is a potential true positive of probable clinical mechanistic relevance, since there is evidence for a relationship between acute myeloid leukaemia and gene mutations associated with hemochromatosis [42]. The *Max Cover* (*α*,*β*)*-FS* approach, therefore, was not only successful in identifying most of the concordant probes identified by the other analytical approaches, but also identified additional discordant probes of probable relevance.

## 4. Discussion

This study demonstrates that, when expression changes are modest, the choice of normalization and analysis algorithms for Illumina microarray data can have a substantial effect on identification of altered genes and pathways. This may considerably influence decisions about which molecular systems are selected for further investigation and the direction of future research. The main findings are summarized here and discussed in detail below.

- The *No Normalization* strategy may be poorly suited to discovery-driven research.
- Background correction in GenomeStudio generally led to a reduction in the size of probe sets, but did not affect percentage concordance.
- Of the four Illumina GenomeStudio normalization strategies, *Cubic Spline*, *Quantile* and *Rank Invariant* generally gave comparable outcomes for a particular analytical approach, although performance sometimes varied between the datasets. (*Average* did not perform as well, particularly in the brain dataset.)
- Different analytical approaches (GenomeStudio, GeneSpring, *Max Cover* (*α*,*β*)*-FS*) often generated quite different probe sets that were enriched for different pathways, even when using the same normalization strategy.
- Most combinations of normalization strategy and analytical approach compared favourably with the Bioconductor tools *lumi* and *limma*.

The results showed that optimal combinations of normalization strategies and analytical approaches may vary considerably for different datasets in ways that are not always readily predictable. It was not possible to choose one combination that works best all the time. It is important to test combinations of different approaches to improve robustness and, wherever feasible, to validate outcomes by alternative methods.

While a number of studies have evaluated the performance of the Illumina microarray platform compared to other platforms [7,8,9,10,11], there is little information on how the choice of different normalization and analysis approaches for Illumina data affects outcomes. One previous study investigated a range of different normalization strategies specifically using Illumina human microarray data [15], but incorporated various approaches only available through R/Bioconductor packages and did not assess the effects of different combinations of normalization strategy and analytical approach on pathway outcomes. Understanding the effects of using different approaches may be particularly important when analysing data involving subtle expression changes, where even minor differences in the scaling of raw data may lead to data adjustments that are comparable in size to the expression changes being investigated. This factor, combined with differences in the way that data are subsequently compared, could considerably influence the identification of “differentially expressed” genes.

The findings suggest that some form of normalization should be applied, since the *No Normalization* strategy resulted in the generation of very small probe sets, as would be expected, since data not adjusted for technical variation are likely to show high variability. All four normalization strategies (*i.e.*, *Average*, *Cubic Spline*, *Quantile* and *Rank Invariant*) performed well in most analyses. Except in the case of *Cubic Spline* and *Quantile* normalization, the high degree of concordance observed when using these methods is unlikely to be an artefact arising from similarities in the normalization procedures, as the various strategies use fundamentally different mathematical approaches. 

The variability in probe sets generated by different normalization strategies makes it difficult to recommend one that will invariably perform best for any analytical approach and any dataset. For optimal performance for discovery-driven research, we would suggest comparing all four normalization strategies for each new investigation.

Similarly, it was shown that the same normalization strategy can give very different outcomes when used with different analytical approaches. The most accessible analysis software for Illumina users, the proprietary Illumina GenomeStudio, does well in that most of the probes it identified were concordant with the other methods investigated, including the Bioconductor tools *lumi* and *limma*. However, it typically generated substantially smaller probe sets than the other approaches and so may miss a considerable number of important genes in some datasets. GeneSpring generally identified a higher total proportion of fully and partially concordant probes than other approaches. *Max Cover* (*α*,*β*)*-FS* also generally identified high numbers of fully and partially concordant probes and in addition found further probes not identified by other approaches. While some of these additional probes may be false positives, some appear to represent real changes that help identify additional pathways of biological relevance.

*Max Cover* (*α*,*β*)*-FS* has a very different mathematical basis from the analytical approaches based on statistical significance (GenomeStudio, GeneSpring). While this may decrease the numbers of fully concordant probes in comparisons of these approaches, those probes that are jointly identified by such very different methods are more likely to represent robust findings. Therefore, in addition to recommending that more than one normalization strategy be used, the use of more than one analytical approach, preferably not restricted solely to statistical testing, is also recommended. 

The findings also suggest that important pathways and processes may be overlooked if only one approach is used to analyse differential gene expression, further highlighting the need for using combinations of approaches. As there were often considerable differences between the findings for the two datasets, it is not possible to recommend a single combination of normalization strategy and analytical approach that will be optimal in all circumstances, particularly since the two datasets examined here were relatively similar (different tissues from the same model) and differences may be even greater for other datasets. Due to individual variability, there may be no “correct” approach—statistical methods may do better in some sample sets, in particular those with low variability, but may miss useful findings in others. The optimum combination of methods will also vary depending on whether the main aim is to minimize false positives, as required for class prediction aimed at biomarker discovery, or to maximize true positives and minimize false negatives, as required in class comparison or class discovery studies.

The use of multiple approaches to identify robust changes differs from more conventional microarray analysis pipelines that utilize multiple testing corrections to avoid false positive findings; however, in this case, we believe it is appropriate. This point is particularly relevant since the GenomeStudio software does not allow the removal of low signal probes (representing non-expressed genes) prior to differential expression analysis, thereby increasing the burden of multiple testing. In addition, *Max Cover* (*α*,*β*)*-FS* appears to yield important findings of biological relevance; yet, as a non-statistical approach, it is not amenable to multiple testing correction. It would be unfortunate if this valuable complementary method were to be discarded solely on these grounds.

Reference RNA that contains many transcripts of known concentration would be ideal for testing the ability of different approaches to identify true positives and true negatives. However, as far as we could determine, reference RNA of this type is not commercially available. Instead, experiments seeking to evaluate reproducibility across platforms or across processing and analysis approaches have relied on either titrations of two distinct RNA reference samples (e.g., universal RNA and brain RNA) [7] or “spike-in” experiments, where genes normally absent from the genome under investigation (e.g., bacterial or viral genes) are added at known concentrations [17,18]. While such experiments provide RNA pools where relative levels of certain transcripts are known *a priori*, they generally result in relatively large fold differences between samples. As our study specifically focused on datasets with small fold changes, it was not feasible to adopt a similar approach in our evaluation. 

Similarly, the small magnitude of most of the fold changes under investigation made it infeasible to test many results by quantitative reverse transcription PCR (qRT-PCR), which is often employed as a method for validating microarray findings. Other groups have reported that fold changes of less than 1.4 by microarray generally show poor correlation with qRT-PCR [43]. While we have used this technique previously to successfully validate some of the most robust findings in the brain dataset [29] and heart dataset (Johnstone *et al.*, unpublished data), these specific changes exceeded the 1.4-fold threshold. 

Therefore, one important limitation of the study is that the accuracy of different outcomes could not be directly assessed and using concordance to estimate accuracy may not always give a true picture. While outside the scope of the present study, future research could compare microarray results obtained using different analytical approaches with other sensitive multiplex or transcriptome-wide technologies, such as other array platforms, RNA-seq, NanoString or Fluidigm. However, it is important to note that human and other technical errors will affect quantitative differential expression analysis by any method, and any comparison requires that the analysis methods for the comparison technology have been shown to be accurate for low fold changes. As far as we are aware, this has not yet been achieved. For example, RNA-seq is biased towards high expression transcripts, so the accuracy of differential expression determinations will vary depending on the expression levels of the transcript. 

Identifying probes as differentially expressed by two or three different methods and detecting enrichment of molecular pathways of strong biological relevance provides some assurance in the accuracy of the findings, as noted above. Also, the strong performance of particular approaches with respect to identifying concordant probes for two different datasets suggests a high degree of reliability in generating robust probe sets. 

Some of the issues addressed in this study may be circumvented by using larger replicate numbers or more sophisticated analytical algorithms. However, even when using high end software packages, consideration should still be given as to how different computational approaches affect study outcomes for different datasets [15]. Furthermore, many researchers lack the expertise to use tools such as *lumi* [12] or *limma* [13] or other Bioconductor packages, which require knowledge of the R programming language. For these reasons, it is important to understand and take into account the strengths and limitations of Illumina-recommended protocols, such as GenomeStudio and GeneSpring, for normalization and differential expression analysis. The findings should not be interpreted as implying that the Illumina platform and software give invalid or incorrect results. Probe sets identified by the GenomeStudio approach showed a high level of concordance with the other approaches, irrespective of the dataset and normalization strategies. However, our findings do indicate that outcomes can be further improved by using other analytical approaches. 

Most of the issues raised here are not unique to the Illumina platform. On other platforms, normalization and analysis methods can affect precision, sensitivity and other factors, and a method that is optimal in one context may be problematic in others [8,44]. The bead technology of Illumina arrays provides strong internal technical replication that is likely to be particularly important for detecting small expression changes. The platform successfully identified gene expression changes of high probable relevance in our study and appears likely to be appropriate for studies involving small expression changes, provided suitable normalization and analytical strategies are used.

## 5. Conclusions

In conclusion, this study has identified a range of potential pitfalls in analysing low expression fold-change datasets and highlights the need for future studies using reference datasets of known positives. While these issues are particularly relevant for datasets where expression changes are expected to be modest, many similar considerations are likely to apply for datasets where most gene expression changes are large, since these will usually still also contain some genes of biological interest with small expression changes. Important effects may be overlooked if there is a habitual routine of using only one approach to investigate all array datasets in a laboratory or commercial testing service. The findings presented here provide guidelines to help researchers optimize outcomes when working with datasets involving small expression changes. Notably, it is proposed that microarray data should be routinely subjected to alternative normalization and analysis procedures and comparisons made between these to obtain more robust gene lists and pathway identifications.

## Appendix

**Table S1:** Pairwise comparisons of probe sets generated by different normalization strategies. Data are presented as the number of overlapping probes between each possible pairwise comparison of the five normalization strategies, with the percentage overlaps for the same comparisons in parentheses.

**Heart–GenomeStudio**								
	No Norm	Average		Cubic Spline		Quantile		Rank Invariant
No Norm	X	548 (58.0)		468 (52.8)		486 (48.6)		510 (48.0)
Average	548 (96.1)	X		775 (87.5)		845 (84.6)		872 (82.1)
Cubic Spline	468 (82.1)	775 (82.0)		X		873 (87.4)		836 (78.7)
Quantile	486 (85.3)	845 (89.4)		873 (98.5)		X		945 (89.0)
Rank Invariant	510 (89.5)	872 (92.3)		836 (94.4)		945 (74.2)		X
**Heart–GeneSpring**								
	No Norm	Average		Cubic Spline		Quantile		Rank Invariant
No Norm	X	821 (56.6)		696 (45.1)		694 (45.1)		685 (45.5)
Average	821 (83.4)	X		1,241 (80.5)		1,241 (80.6)		1,224 (81.3)
Cubic Spline	696 (70.7)	1,241 (85.5)		X		1,509 (98.1)		1,373 (91.2)
Quantile	694 (70.5)	1,241 (85.5)		1,509 (97.9)		X		1,374 (91.2)
Rank Invariant	685 (69.6)	1,224 (84.4)		1,373 (89.0)		1,374 (89.3)		X
**Heart–Max Cover (*α*,*β*)-*FS***								
	No Norm	Average		Cubic Spline		Quantile		Rank Invariant
No Norm	X	870 (56.6)		759 (46.2)		752 (45.9)		742 (46.5)
Average	870 (79.5)	X		1,297 (78.9)		1,288 (78.6)		1,268 (79.5)
Cubic Spline	759 (69.3)	1,297 (84.4)		X		1,616 (98.6)		1,456 (91.3)
Quantile	752 (68.7)	1,288 (83.8)		1,616 (98.3)		X		1,456 (91.3)
Rank Invariant	742 (67.8)	1,268 (82.5)		1,456 (88.6)		1,456 (88.8)		X
**Brain–GenomeStudio**								
	Average		Cubic Spline		Quantile		Rank Invariant	
Average	X		44 (33.1)		44 (26.3)		43 (33.9)	
Cubic Spline	44 (83.0)		X		132 (79.0)		104 (81.9)	
Quantile	44 (83.0)		132 (99.2)		X		109 (85.8)	
Rank Invariant	43 (81.1)		104 (78.2)		109 (65.3)		X	
**Brain–GeneSpring**								
	Average		Cubic Spline		Quantile		Rank Invariant	
Average	X		145 (41.8)		145 (40.8)		111 (34.4)	
Cubic Spline	145 (62.8)		X		341 (96.1)		258 (79.9)	
Quantile	145 (62.8)		341 (98.3)		X		259 (80.2)	
Rank Invariant	111 (48.1)		258 (74.4)		259 (73.0)		X	
**Brain–Max Cover (*α*,*β*)-*FS***								
Average	Average		Cubic Spline		Quantile		Rank Invariant	
Cubic Spline	X		213 (35.1)		209 (34.6)		149 (27.2)	
Quantile	213 (49.0)		X		588 (97.4)		406 (74.2)	
Rank Invariant	209 (48.0)		588 (96.9)		X		406 (74.2)	
Average	149 (34.3)		406 (66.9)		406 (67.2)		X	

**Table S2:** Pairwise comparisons of probe sets generated by different normalization strategies, with multiple testing correction. Data are presented as the number of overlapping probes between each possible pairwise comparison of the five normalization strategies, with the percentage overlaps for the same comparisons in parentheses.

Heart–GenomeStudio					
	No Norm	Average	Cubic Spline	Quantile	Rank Invariant
No Norm	X	17 (34.0)	16 (28.1)	16 (26.2)	17 (21.5)
Average	17 (100)	X	47 (82.5)	48 (78.7)	49 (62.0)
Cubic Spline	16 (94.1)	47 (94.0)	X	57 (93.4)	57 (72.2)
Quantile	16 (94.1)	48 (96.0)	57 (100)	X	60 (75.9)
Rank Invariant	17 (100)	49 (98.0)	57 (100)	60 (98.4)	X

**Table S3:** Pairwise comparisons of probe sets generated by different normalization strategies, with no background correction. Data are presented as the number of overlapping probes between each possible pairwise comparison of the five normalization strategies, with the percentage overlaps for the same comparisons in parentheses.

**Heart–GenomeStudio**								
	No Norm	Average		Cubic Spline		Quantile		Rank Invariant
No Norm	X	689 (50.6)		621 (48.6)		626 (47.9)		648 (43.2)
Average	689 (92.7)	X		1,146 (89.7)		1,183 (90.4)		1,242 (82.9)
Cubic Spline	621 (83.6)	1,146 (80.2)		X		1,249 (95.5)		1,185 (79.1)
Quantile	626 (84.3)	1,183 (86.9)		1,249 (97.7)		X		1,225 (81.7)
Rank Invariant	648 (87.2)	1,242 (91.3)		1,185 (92.7)		1,225 (93.7)		X
**Brain–GenomeStudio**								
	Average		Cubic Spline		Quantile		Rank Invariant	
Average	X		61 (33.0)		61 (30.3)		56 (45.2)	
Cubic Spline	61 (82.4)		X		181 (90.0)		113 (91.1)	
Quantile	61 (82.4)		181 (97.8)		X		114 (91.9)	
Rank Invariant	56 (75.7)		113 (61.1)		114 (56.7)		X	

**Table S4:** Comparison of probe sets generated by different combinations of the normalization strategy and analytical approach, with probe sets generated by the Bioconductor packages, *lumi* and *limma*.

Heart Dataset	*vs*. Lumi (2,239 probes)			*vs*. Limma (2,107 probes)		
	Number Concordant	Number Discordant	% Concord	Number Concordant	Number Discordant	% Concord
**GenomeStudio**						
No Norm	551	19	96.7	535	35	93.9
Average	935	10	98.9	922	23	97.6
Cubic Spline	884	2	99.8	876	10	98.9
Quantile	997	2	99.8	989	10	99.0
Rank Invariant	1,060	2	99.8	1,051	11	99.0
**GeneSpring**						
No Norm	828	156	84.1	820	164	83.3
Average	1,371	80	94.5	1,366	85	94.1
Cubic Spline	1,512	30	98.1	1,508	34	97.8
Quantile	1,507	32	97.9	1,507	32	97.9
Rank Invariant	1,460	46	96.9	1,458	48	96.8
**Max Cover (*α*,*β*)-*FS***						
No Norm	900	195	82.2	885	210	80.8
Average	1,382	155	89.9	1,365	172	88.8
Cubic Spline	1,532	112	93.2	1,522	122	92.6
Quantile	1,530	109	93.3	1,517	122	92.6
Rank Invariant	1,480	115	92.8	1,464	131	91.8
**Brain Dataset**	***vs*. Lumi (488 probes)**			***vs*. Limma (420 probes)**		
	Number Concordant	Number Discordant	% Concord	Number Concordant	Number Discordant	% Concord
**GenomeStudio**						
No Norm	1	0	100	1	0	100
Average	47	6	88.7	43	10	81.1
Cubic Spline	128	5	96.2	116	17	87.2
Quantile	157	10	94.0	142	25	85.0
Rank Invariant	118	9	92.9	107	20	84.3
**GeneSpring**						
No Norm	1	3	25.0	1	3	25.0
Average	161	70	69.7	151	80	65.4
Cubic Spline	313	34	90.2	309	38	89.0
Quantile	316	39	89.0	311	44	87.6
Rank Invariant	271	52	83.9	261	62	80.8
**Max Cover (*α*,*β*)-*FS***						
No Norm	1	11	8.3	1	11	8.3
Average	168	267	38.6	160	275	36.8
Cubic Spline	298	309	49.1	280	327	46.1
Quantile	299	305	49.5	283	321	46.9
Rank Invariant	249	298	45.5	240	307	43.9
